# Long-term accumulation of diphenylarsinic acid in the central nervous system of cynomolgus monkeys

**DOI:** 10.1007/s00204-016-1928-z

**Published:** 2017-01-25

**Authors:** Tomoyuki Masuda, Kazuhiro Ishii, Yasuo Seto, Tomoko Hosoya, Ryuta Tanaka, Tomohiro Nakayama, Nobuaki Iwasaki, Yasuyuki Shibata, Akira Tamaoka

**Affiliations:** 1 Department of Neurology, Faculty of Medicine, University of Tsukuba, Ibaraki, 305-8575 Japan; 2 Doctoral and Master’s Programs in Kansei, Behavioral and Brain Sciences, Graduate School of Comprehensive Human Sciences, University of Tsukuba, Ibaraki, 305-8577 Japan; 3 Department of Neurobiology, Faculty of Medicine, University of Tsukuba, Ibaraki, 305-8577 Japan; 4 Third Department of Forensic Science, National Research Institute of Police Science, Chiba, 277-0882 Japan; 5 Department of Child Health, Faculty of Medicine, University of Tsukuba, Ibaraki, 305-8575 Japan; 6 Department of Pediatrics, Ibaraki Prefectural University of Health Sciences, Ibaraki, 300-0331 Japan; 7 Center for Environmental Measurement and Analysis, National Institute for Environmental Studies, Ibaraki, 305-8506 Japan

**Keywords:** LC–MS, *Macaca fascicularis*, Cerebrum, Cerebellum, Primate, Phenyl arsenic compound

## Abstract

Diphenylarsinic acid (DPAA) is an organic arsenic compound used for the synthesis of chemical weapons. We previously found that the residents of Kamisu city in Ibaraki Prefecture, Japan, were exposed to DPAA through contaminated well water in 2003. Although mounting evidence strongly suggests that their neurological symptoms were caused by DPAA, the dynamics of DPAA distribution and metabolism after ingestion by humans remain to be elucidated. To accurately predict the distribution of DPAA in the human body, we administrated DPAA (1.0 mg/kg/day) to cynomolgus monkeys (*n* = 28) for 28 days. The whole tissues from these monkeys were collected at 5, 29, 170, and 339 days after the last administration. The concentration of DPAA in these tissues was measured by liquid chromatography–mass spectrometry. We found that DPAA accumulated in the central nervous system tissues for a longer period than in other tissues. This finding would extend our knowledge on the distribution dynamics and metabolism of DPAA in primates, including humans. Furthermore, it may be useful for developing a treatment strategy for patients who are exposed to DPAA.

## Introduction

Seaweed contains a large number of organic arsenic compounds, including non-toxic compounds, and many toxic compounds are synthesized as pharmaceutical and chemical warfare agents. Diphenylarsinic acid (DPAA) is an organic arsenic compound that was used for the synthesis of chemical weapons during World War II (Kurata 1980). In 2003, several patients with illnesses of unknown origin were reported in Ibaraki Prefecture, Japan. Organic arsenic compounds that were found in the wells of Kamisu city in Ibaraki were identified as the cause of these symptoms (Ishii et al. 2004). Contaminated water from one of the wells contained 4.5 mg As/L of DPAA, which is approximately 450 times higher than the concentration permitted by the drinking water quality standards in Japan (Kinoshita et al. 2005; Shibata et al. 2005). A total of 157 residents who were exposed to DPAA presented with progressive cerebellar and brain stem symptoms, including nystagmus, dizziness, ataxic gait, tremors, myoclonus and dysarthria, along with temporal and occipital lobe symptoms, including memory impairment, sleep disturbance, and visual disorders, as well as cerebral atrophy and mental retardation in children. The patients have been receiving regular medical checkups for more than 10 years.

Although mounting evidence strongly suggests that these neurological symptoms were caused by DPAA (Ishii et al. 2004), our knowledge about the in vivo distribution and effects of DPAA remains limited. The effects of DPAA in higher vertebrates have been demonstrated using rodents. The oral administration of DPAA to mice led to higher accumulation of DPAA in the brain than in the liver (Ozone et al. 2010). Furthermore, DPAA has been reported to damage the Purkinje cells and cause behavioral impairment in mice (Ozone et al. 2010; Umezu et al. 2012). In rats, the oral administration of DPAA led to a higher accumulation of DPAA in the brain and pancreas in comparison to inorganic arsenic compounds (Naranmandura et al. 2009), as well as behavioral impairment (Negishi et al. 2013). We previously administered DPAA to rats and established a rodent model with the same acute cerebellar and brain stem symptoms as those of patients in Kamisu (Masuda and Ishii, unpublished observation). However, their symptoms lasted for a shorter period compared to those of the patients (Masuda and Ishii, unpublished observation). Several studies have also shown that the distribution and metabolism of arsenic compounds are different among rodents and primates (Vahter and Marafante 1985; Vahter et al. 1995; Aposhian 1997). Therefore, it is difficult for us to predict the pattern of DPAA accumulation in the human central nervous system (CNS) solely based on the data from rodent studies. Thus, we decided to use primates to examine the accurate distribution of DPAA in the human CNS. The only primate study in relation to DPAA investigated the DPAA concentrations in the blood, urine, and feces of primates after oral administration (Kobayashi et al. 2008).

Previously, DPAA and its related metabolites have been identified in biological and environmental samples using liquid chromatography (LC) coupled with induced coupled plasma ionization mass spectrometry (ICP–MS), in which they were detected as arsenic atom-containing compounds (Kobayashi et al. 2008; Hempel et al. 2009). In the present study, we adopted isotope dilution LC–mass spectrometry (LC–MS). Internal standard compounds of DPAA and their metabolites labeled with ^13^C were added to the tissue samples. Unlabeled target phenyl arsenic compounds and their corresponding isotope-labeled congeners were extracted and subjected to LC–MS. Isotope dilution LC–MS is extremely useful because it can provide exact compensation of extraction recovery from biological samples and efficient mass spectrometric ionization.

In the present study, we used cynomolgus monkeys to predict the distribution and metabolism of DPAA in humans. We measured the concentration of DPAA in various tissue samples by LC–MS at 5–339 days after the last administration of DPAA.

## Materials and methods

### Arsenic

Phenyl arsenic compounds [DPAA (>99.0%), DPMAO (diphenylmethylarsine oxide: >99.0%), phenylmethylarsinic acid (PMAA: >99.0%), phenyldimethylarsine oxide (PDMAO: >99.0%), and phenylarsonic acid (PAA: >99.0%)] were purchased from Tri Chemical Laboratories (Yamanashi, Japan) and stored at 4 °C in the dark. Stable radioactive isotopes (^13^C-DPAA, ^13^C-DPMAO, ^13^C-PMAA, ^13^C-PDMAO, and ^13^C-PAA: >95.0%) were produced by Hayashi Pure Chemical Industries (Osaka, Japan).

### Animals

Twenty-eight cynomolgus monkeys (*Macaca fascicularis*) were purchased from the HAMRI CO., LTD. (Ibaraki, Japan). The average weights of the male and female monkeys were 3.44 kg (*n* = 14) and 2.81 kg (*n* = 14), respectively. The administration of DPAA to the monkeys and tissue sampling were performed at LSI Medience Corporation (Tokyo, Japan). All of the experiments were conducted in accordance with the Guidelines for Proper Conduct of Animal Experiments by the Science Council of Japan.

### Animal experiments and the sampling of tissues and body fluids

DPAA was dissolved in distilled water at a concentration of 10 mg/mL and the pH was adjusted to 7.0 with 1 N NaOH. It was then administrated through a nasogastric tube at a dose of 1.0 mg/kg/day. This concentration was determined based on the approximate levels of human exposure in Kamisu. After the repeated administration of DPAA for 28 days, the monkeys were divided into four groups. Monkeys in the first group (*n* = 6), second group (*n* = 6), third group (*n* = 8) and fourth group (*n* = 8) were euthanized at 5, 29, 170, and 339 days after the last administration, respectively.

After their euthanization, the following tissue types were excised and collected: eight types derived from the CNS, the sciatic nerves, three types from the emunctories, eight types from the urogenital system, seven types from the digestive system, four types from the lymph system, four types from the exocrine system, three types from the endocrine system, two types from the respiratory system, two types from the muscular system, two types from the sense organs, and two types from the blood–vascular system (see Tables). These samples were then frozen in liquid nitrogen and stored at −80 °C. Also, four types of body fluids (cerebrospinal fluid, bile, hemocyte, and serum) were collected and stored at −80 °C.

### Extraction of phenyl arsenic compounds

20% of the homogenates of tissue samples were prepared in 50 mM ammonium acetate solution (Wako Pure Chemical Industries, Ltd., Osaka, Japan) using a Teflon homogenizer (Ikemoto Scientific Technology Co., Ltd., Tokyo, Japan). Then, these homogenized samples, as well as the cerebrospinal fluid (CSF) and bile samples were ultra-centrifuged at 105,000×*g* at 4 °C and the supernatants were obtained. The collected blood was centrifuged at 3000×*g* for 10 min and separated into hemocytes and serum. The hemocytes were washed three times with 50 mM of Tris-buffered saline (pH 7.4). Thereafter, 10 mM of Tris–HNO_3_ buffer was added to the hemocyte solution and centrifuged at 15,000×*g* for 30 min.

Next, 0.1 mL of bovine serum albumin (80 mg/mL; Takara Bio Inc., Shiga, Japan), 1 mL of 4 M NaOH, and 0.9 mL of H_2_O were added to 0.1 g of the samples prepared above. Then, stable radioactive isotopes ^13^C-DPAA, ^13^C-DPMAO, ^13^C-PMAA, ^13^C-PDMAO, and ^13^C-PAA (each 100 ppb) were added to the mixed solution and incubated at 90 °C for 3 h. After adding diethyl ether (Wako Pure Chemical Industries, Ltd.: >99.5%), the mixed solution was centrifuged for 5 min to extract phenyl arsenic compounds with diethyl ether. The diethyl ether was then removed from the phenyl arsenic compounds using a stream of dry N_2_. After the addition of nitric acid (Wako Pure Chemical Industries, Ltd.), the phenyl arsenic compounds were completely dissolved in 10 mL of H_2_O.

### Analysis of phenyl arsenic

Shiseido Nanospace SI-2 (Shiseido Co., Ltd., Tokyo, Japan) and TSQ Quantum Ultra (Thermo Fisher Scientific Corp., CA, USA) LC–MS instruments were used. Chromatographic separation was achieved using two reversed-phase columns (Imtakt Unison UK-C18; 150 × 3.0 mm i.d., 3 µm thickness, Kyoto, Japan; Imtakt Unison UP-Phenyl, 150 × 3.0 mm i.d., 3 µm thickness) at 40 °C. 50 µL of sample solution was injected. In both columns, elution was performed using a linear gradient of 0.1% formic acid (Wako Pure Chemical Industries, Ltd.: 99.0%) in water [A] with 0.1% formic acid in methanol–water (1:9, v/v) [B] as follows: 95% [A]: 5% [B] (5-min hold), 95% [A]: 5% [B] to 100% [B] (5–10 min), 100% [B] (5-min hold), 100% [B] to 95% [A]: 5% [B] (20–10 min), 95% [A]: 5% [B] (14-min hold). The flow rate was maintained at 0.25 mL/min. Electrospray ionization was performed in positive ionization mode. The [M + H]^+^ions of the targeted compounds (DPAA, ^13^C-DPAA, DPMAO, ^13^C-DPMAO, PMAA, ^13^C-PMAA, PDMAO, ^13^C-PDMAO, PAA, ^13^C-PAA) were selected as the precursor ions. Selected reaction monitoring was used to quantify each compound. The precursor and product ions were monitored. The collision energies of the compounds were as follows: *m*/*z* 263 > 152, 30 eV for DPAA; *m*/*z* 275 > 158, 30 eV for ^13^C-DPAA; *m*/*z* 261 > 154, 35 eV for DPMAO; *m*/*z* 273 > 160, 35 eV for ^13^C-DPMAO; *m*/*z* 203 > 77, 21 eV for PAA; *m*/*z* 209 > 83, 21 eV for ^13^C-PAA; *m*/*z* 201 > 77, 35 eV for PMAA; *m*/*z* 207 > 83, 35 eV for ^13^C-PMAA; *m*/*z* 199 > 169, 25 eV for PDMAO; and *m*/*z* 205 > 175, 25 eV for ^13^C-PDMAO. To determine the compounds with greater precision, we monitored the other product ions as well: *m*/*z* 203 > 185 for PAA; *m*/*z* 201 > 94 for PMAA; *m*/*z* 199 > 77 for PDMAO. The MS parameters for the analysis were as follows: spray voltage, 4000 V; sheath gas (N_2_) pressure, 6 Pa; auxiliary gas (N_2_) pressure, 0.5 Pa; capillary temperature, 230 °C; vaporizing temperature, 450 °C; tube lens offset, 100 V; collision gas (Ar) pressure, 0.2 Pa. Data acquisition and instrument control were performed using the Xcalibur software program (Thermo Fisher Scientific Corp.).

Also, a phenyl column was used for the qualitative confirmation of the target compounds detected in the C18 column analysis, and to avoid the false-positive detection of the target compounds in the C18 column analysis. The retention times, peak widths at half height, and the limit of detection (LOD, signal-to-noise ratio = 3) of the phenyl arsenic compounds in aqueous solution on the C18 column analysis were 14.2 min, 0.086 min, and 14 pg/mL, respectively, for DPAA; 13.8 min, 0.066 min, and 6.2 pg/mL for DPMAO; 12.4 min, 0.086 min, and 140 pg/mL for PAA; 12.5 min, 0.065 min, and 130 pg/mL for PMAA; and 11.8 min, 0.097 min, and 62 pg/mL for PDMAO. Similarly, the values for the phenyl arsenic compounds that were run on the phenyl column were 14.3 min, 0.086 min, and 25 g/mL, respectively, for DPAA; 14.2 min, 0.063 min, and 18 pg/mL for DPMAO; 11.4 min, 0.233 min, and 910 pg/mL for PAA; 12.4 min, 0.106 min, and 83 pg/mL for PMAA; and 12.2 min, 0.064 min, and 130 pg/mL for PDMAO. The LOD values for the tissue extract samples were higher than those for aqueous solution, depending on the tissue sample matrix. This was due to interference from the matrix, which typically suppresses the ionization of the target compounds and prevents the detection of peaks with overlapping matrix components. In the C18 column analysis, the relative intra-day deviations (*n* = 4) of the peak area ratios on the product ion spectra for arsenic compounds (20 ng/mL) to those for corresponding ^13^C-labelled ones (5 ng/mL; internal standards) were 0.9, 1.8, 2.7, 5.5, and 3.5% for DPAA, DPMAO, PAA, PMAA and PDMAO, respectively. The relative intra-day deviations in the phenyl column analysis (*n* = 5) were 3.6, 1.3, 7.9, 4.2, and 3.7%, respectively.

### Data analysis

To calculate the half-life (*T*_1/2_; the time required for the concentration to decrease to half its initial value) of DPAA, we plotted the DPAA concentrations (at 5 and 29 days after the last administration of DPAA) in each of tissue and body fluid sample against the concentration values on the logarithmic scale. The half-lives were calculated based on the slope of the decay lines (slope = −*k*/2.303, *T*_1/2_ = 0.693/*k*).

### Statistical analyses and preparation of approximate graph curves

Tukey’s method was used for multiple group comparisons. The JMP software program (version 5.12-J, SAS Institute Inc., NC, USA) was used to perform the statistical analyses. *P* values of <0.05 were considered to indicate statistical significance. The JMP software program (version 5.12-J) was also used to perform a curvilinear regression analysis, which allowed us to approximate the quadratic curves.

## Results

### Liquid chromatography–mass spectrometry

**Fig. 1:**
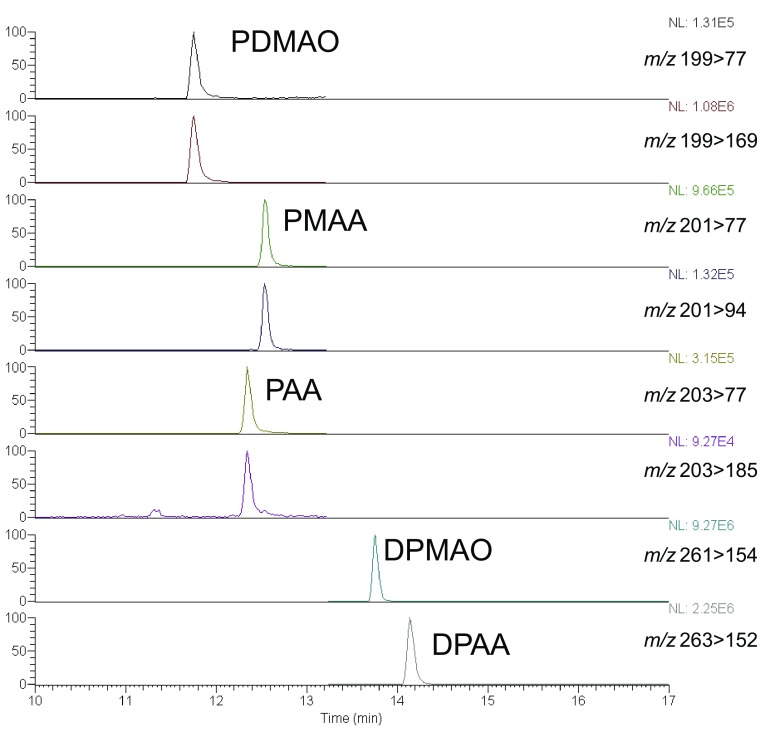
A selected reaction monitoring chromatogram for the phenyl arsenic compounds in aqueous solution. 50 µL of DPAA, DPMAO, PAA, PMAA, and PDMAO solution (each 33.3 ng/mL) was injected into the LC–MS system fitted with a C18 column. The 100% ionic intensities and mass transitions are shown on the right. (Color figure online)

DPAA and its related metabolites were identified in tissue samples by LC–MS using the isotope dilution method. These phenyl arsenic compounds were well separated in LC conditions using both C18 and phenyl columns. The [M + H]^+^ ions of the target compounds were carefully observed by electrospray ionization. 50 µL of the sample solution was injected into the LC–MS system to achieve optimal detection sensitivity. Imtakt Unison columns enabled large-volume injection without compromising the peak resolution. During selected reaction monitoring (SRM), the product ions and collision energies were selected by optimizing the conditions in the product ion scan spectra of the target precursor ions. A typical SRM chromatogram for the control phenyl arsenic compounds is shown in Fig. 1. Calibration curves were prepared for the peak area ratios of the target phenyl arsenic compounds of the corresponding ^13^C-labeled internal standards on the respective SRM chromatograms. We first used a C18 column to determine the target compounds in the samples. If positive peaks were observed on SRM, the presence of the corresponding product ion peaks was tested for PAA, PMAA, and PDMAO to provide definitive confirmation of their presence, because these highly hydrophilic compounds typically suffer from severe interference from the biological matrix components in sample extracts during LC separation and MS detection.

### Quantitative analysis of DPAA in the tissues of cynomolgus monkeys

Twenty-eight cynomolgus monkeys were exposed to DPAA for 28 days at a dose of 1.0 mg DPAA/kg/day. After the oral administration of DPAA, the monkeys were divided into four groups (each group contained 6–8 monkeys), which were euthanized at 5, 29, 170, and 339 days after the last administration of DPAA. The whole tissues and body fluids were collected after euthanasia (Table 1).

**Table 1:** DPAA concentration in the tissues of cynomolgus monkeys after oral administration of DPAA

	DPAA (ng As/g) after administration				Half-life of DPAA (day)
	Days 5 (average ± SEM)	Days 29 (average ± SEM)	Days 170 (average ± SEM)	Days 339 (average ± SEM)	
Central nervous system					
Cervical spinal cord	588 ± 58.9 (6)	183 ± 12.6 (6)	47.0 ± 3.20 (8)	29.4 ± 3.85 (8)	14.4
Thoracic spinal cord	527 ± 39.1 (6)	167 ± 13.5 (6)	48.5 ± 3.69 (8)	27.4 ± 2.22 (8)	14.4
Lumbar spinal cord	633 ± 84.5 (6)	187 ± 18.0 (6)	40.0 ± 4.32 (8)	20.3 ± 2.70 (8)	13.9
Upper brain stem	659 ± 66.4 (4)	222 ± 10.7 (3)	51.8 ± 3.45 (8)	30.2 ± 4.93 (4)	15.4
Intermediate brain stem	686 ± 77.9 (4)	243 ± 8.05 (4)	55.7 ± 4.33 (8)	36.0 ± 8.10 (4)	16.1
Lower brain stem	682 ± 83.6 (4)	216 ± 15.7 (4)	47.4 ± 2.67 (8)	23.0 ± 3.79 (4)	14.7
Upper cerebellum	446 ± 38.0 (4)	115 ± 10.4 (4)	13.3 ± 1.81 (8)	5.90 ± 2.25 (4)	12.4
Lower cerebellum	470 ± 74.0 (4)	124 ± 9.05 (4)	13.2 ± 1.85 (8)	7.53 ± 1.68 (4)	12.6
Frontal lobe	517 ± 48.3 (4)	145 ± 12.7 (4)	12.5 ± 1.44 (8)	6.45 ± 1.82 (4)	13.1
Parietal lobe	681 ± 89.0 (4)	199 ± 9.63 (4)	25.9 ± 4.42 (8)	13.6 ± 2.57 (4)	13.9
Temporal lobe	518 ± 49.8 (4)	173 ± 23.6 (4)	13.0 ± 2.12 (8)	6.53 ± 2.47 (4)	15.1
Occipital lobe	830 ± 116 (4)	227 ± 36.3 (4)	37.5 ± 5.34 (8)	20.9 ± 1.60 (4)	12.8
Hippocampus	531 ± 51.9 (4)	128 ± 13.0 (4)	16.6 ± 5.05 (8)	5.12 ± 2.18 (4)	11.7
Striatum	772 ± 116 (4)	254 ± 28.0 (4)	35.9 ± 4.09 (8)	25.2 ± 5.45 (4)	15.1
Cingulate gyrus	493 ± 42.1 (4)	143 ± 23.8 (4)	12.7 ± 2.76 (8)	3.19 ± 0.571 (4)	13.1
Central nervous system (total)	600 ± 21.2 (66)*	181 ± 6.60 (65)*	31.4 ± 1.71 (120)*	18.8 ± 1.48 (72)*	14.4*
Peripheral nervous system					
Sciatic nerve	480 ± 50.0 (6)*	250 ± 23.9 (6)*	2.94 ± 0.427 (8)	0.841 ± 0.174 (8)	25.7*
Emunctories					
Liver	555 ± 106 (6)	16.8 ± 3.09 (6)	0.263 ± 0.0543 (8)	0.0620 ± 0.0327 (8)	4.72
Gall bladder	1041 ± 224 (4)	36.5 ± 9.51 (4)	0.732 ± 0.0969 (8)	0.148 ± 0.0569 (8)	4.92
Kidney	532 ± 60.9 (6)	34.5 ± 4.72 (6)	0.378 ± 0.0381 (8)	0.0341 ± 0.0130 (8)	6.03
Emunctories (total)	1033 ± 188 (20)*	41.6 ± 8.32 (20)*	0.761 ± 0.117 (32)	0.148 ± 0.0302 (31)	5.13
Urogenital system					
Bladder	46.5 ± 11.0 (6)	4.48 ± 0.615 (6)	0.213 ± 0.0368 (8)	0 (8)	7.30
Testicle	21.2 ± 3.83 (3)	1.62 ± 0.140 (3)	0 (4)	0 (4)	6.54
Seminal vesicle	49.3 ± 6.25 (3)	7.19 ± 1.25 (3)	0.0197 ± 0.0187 (4)	0 (4)	8.56
Epididymis	38.2 ± 5.32 (3)	4.54 ± 1.02 (3)	0.0727 ± 0.0416 (4)	0.0328 ± 0.0318 (4)	7.70
Prostate	44.2 ± 8.30 (3)	6.46 ± 1.29 (3)	0.0469 ± 0.0271 (4)	0 (4)	8.66
Ovary	39.1 ± 10.9 (3)	4.62 ± 1.67 (3)	0.182 ± 0.0679 (4)	0 (4)	7.30
Uterus	28.3 ± 3.84 (3)	3.83 ± 2.03 (3)	0 (4)	0 (4)	7.30
Vagina	37.5 ± 3.95 (3)	4.21 ± 1.21 (3)	0.0755 ± 0.0354 (4)	0 (4)	7.30
Digestive system					
Esophagus	29.5 ± 3.98 (6)	2.74 ± 0.411 (6)	0.0905 ± 0.0191 (8)	0.0140 ± 0.0130 (8)	6.93
Stomach	20.6 ± 2.08 (6)	1.93 ± 0.215 (6)	0.0422 ± 0.0169 (8)	0 (8)	7.00
Duodenum	55.5 ± 15.0 (6)	6.68 ± 1.43 (6)	0.0751 ± 0.0176 (8)	0 (8)	7.70
Small intestine	73.0 ± 13.7 (5)	4.05 ± 0.723 (6)	0.0939 ± 0.0186 (8)	0.0377 ± 0.0367 (8)	5.68
Colon	44.5 ± 9.41 (6)	3.61 ± 0.630 (6)	0.109 ± 0.0202 (8)	0 (7)	6.67
Rectal	38.5 ± 9.92 (4)	3.01 ± 0.495 (4)	0.123 ± 0.0213 (8)	0 (6)	6.67
Pancreas	54.6 ± 9.42 (6)	3.14 ± 0.494 (6)	0.0381 ± 0.0186 (8)	0.0147 ± 0.0137 (8)	5.83
Lymph system					
Thymus	54.7 ± 7.15 (6)	3.52 ± 0.772 (6)	0.0917 ± 0.0356 (8)	0 (8)	5.87
Spleen	44.7 ± 4.58 (6)	2.28 ± 0.283 (6)	0.00839 ± 0.00739 (8)	0 (8)	5.55
Submandibular node	40.8 ± 3.89 (6)	3.55 ± 0.401 (6)	0.103 ± 0.0183 (7)	0.0166 ± 0.0156 (6)	6.80
Mesenteric node	49.9 ± 7.18 (4)	3.60 ± 0.686 (4)	0.0845 ± 0.0164 (7)	0 (8)	6.30
Exocrine system					
Parotid gland	41.3 ± 10.6 (6)	2.20 ± 0.156 (6)	0.0511 ± 0.0170 (8)	0 (8)	6.03
Submandibular gland	146 ± 35.2 (6)	13.2 ± 1.94 (6)	0.0396 ± 0.0150 (8)	0 (8)	7.53
Sublingual gland	39.7 ± 9.63 (6)	7.36 ± 1.41 (6)	0.0760 ± 0.0295 (8)	0.0445 ± 0.0215 (8)	9.90
Lacrimal gland	62.1 ± 11.0 (4)	3.35 ± 0.697 (4)	0.101 ± 0.0237 (6)	0.0175 ± 0.0165 (8)	5.68
Endocrine system					
Adrenal gland	37.4 ± 6.65 (6)	4.07 ± 2.49 (6)	0.0542 ± 0.0322 (8)	0 (8)	6.03
Pituitary	28.7 ± 2.82 (6)	3.72 ± 0.424 (6)	0.200 ± 0.0382 (8)	0 (8)	8.06
Thyroid	16.2 ± 3.23 (6)	0.946 ± 0.112 (6)	0 (8)	0.0287 ± 0.0277 (8)	6.03
Respiratory system					
Trachea	20.3 ± 1.46 (6)	4.51 ± 0.750 (6)	1.44 ± 0.168 (8)	0.815 ± 0.119 (8)	10.7
Lung	106 ± 15.6 (6)	10.4 ± 1.36 (6)	0.227 ± 0.0578 (8)	0.0259 ± 0.0163 (8)	7.15
Muscular system					
Femoral muscle	25.9 ± 3.72 (6)	5.94 ± 0.746 (6)	0.109 ± 0.0247 (8)	0 (8)	11.4
Tongue	43.0 ± 6.18 (6)	5.31 ± 0.917 (6)	0.0878 ± 0.0245 (8)	0.0213 ± 0.0203 (8)	7.88
Sense organs					
Eyeball	7.80 ± 2.02 (6)	3.06 ± 0.938 (6)	0.260 ± 0.0529 (8)	0.149 ± 0.0492 (8)	20.4
Skin with hair	49.9 ± 4.02 (6)	16.2 ± 4.27 (6)	1.70 ± 0.410 (8)	0.369 ± 0.255 (8)	12.6
Blood–vascular system					
Heart	48.3 ± 6.20 (6)	6.36 ± 0.740 (6)	0.147 ± 0.0245 (8)	0.00730 ± 0.00630 (8)	8.25
Aorta	41.9 ± 7.96 (4)	5.12 ± 0.765 (4)	1.17 ± 0.262 (8)	0.458 ± 0.0896 (8)	7.97
Other tissues (total)	43.2 ± 2.49 (188)	4.76 ± 0.311 (187)	0.218 ± 0.0298 (255)	0.0643 ± 0.0139 (255)	7.30
Body fluids					
Cerebrospinal fluid	1.78 ± 0.252 (6)	0.233 ± 0.0268 (6)	0 (8)	0 (6)	8.25
Bile	2496 ± 287 (4)	94.5 ± 27.4 (4)	1.67 ± 0.228 (8)	0.377 ± 0.0462 (7)	4.88
Hemocyte	9.03 ± 0.936 (2)	2.05 ± 0.782 (2)	0 (1)	–	10.7
Serum	11.4 ± 2.41 (6)	0.364 ± 0.0458 (6)	0 (8)	0 (8)	4.92

The number between brackets indicates the number of samples examined

*p < 0.0001, compared with other tissues

**Fig. 2:**
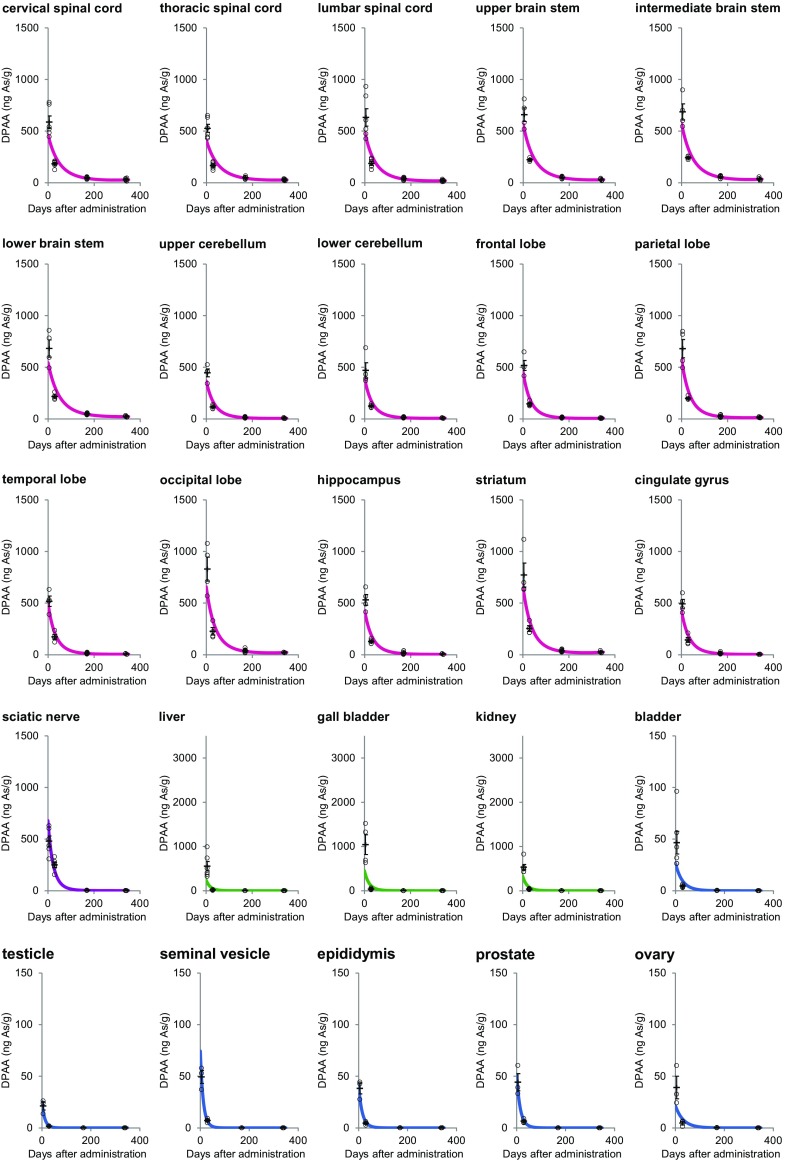

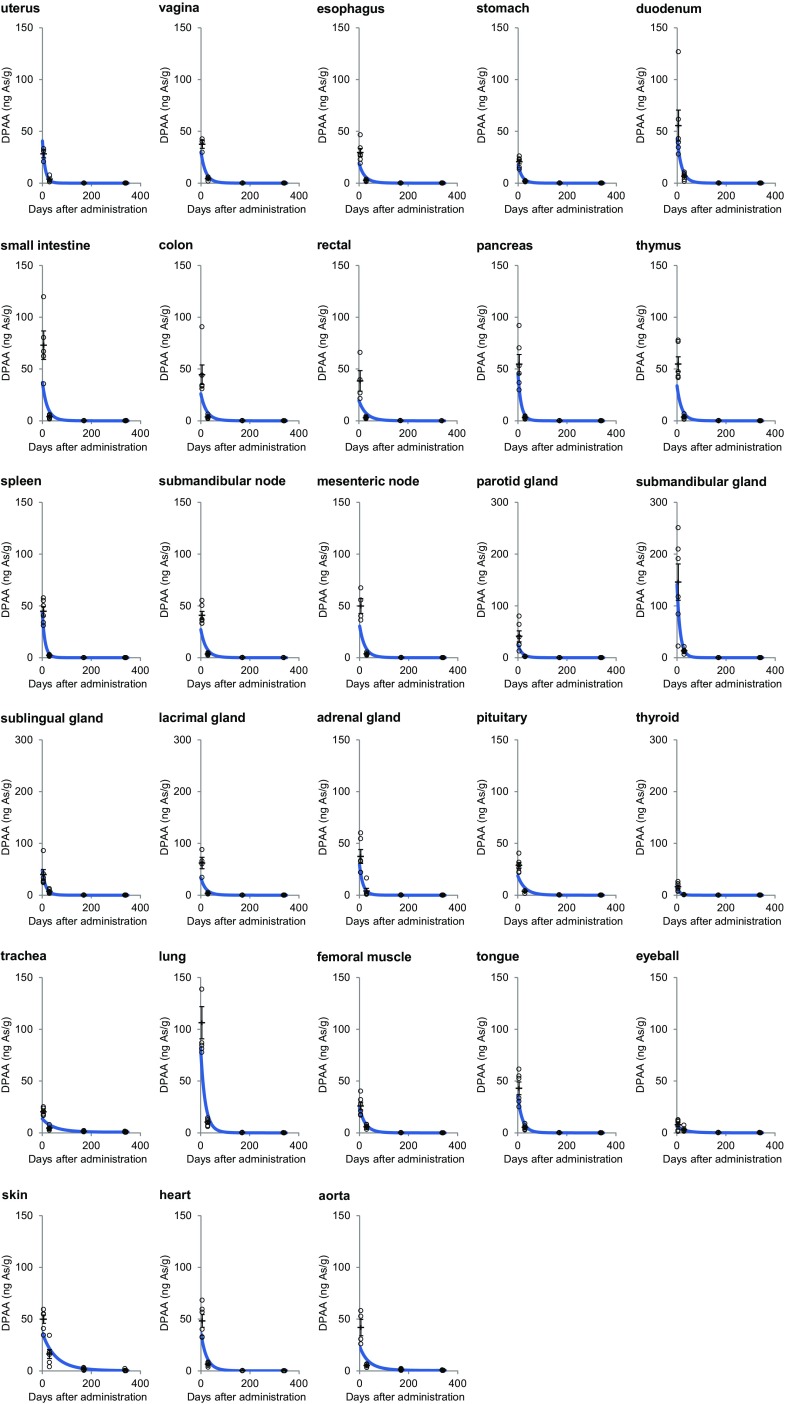
Approximate curves showing the correlation between the days after the last administration of DPAA and the average concentrations of DPAA (ng As/g) in tissues of cynomolgus monkeys after the daily administration of DPAA (1.0 mg/kg/day) for 28 days. (Color figure online)

We measured the concentration of DPAA in the tissues of animals from each group using a LC–MS analysis. The concentrations of DPAA in all of the tissues showed a gradual decrease with time after the last administration (Table 2; Fig. 1). At 5 days after the last administration, we found that the average concentrations of DPAA in the tissues derived from the CNS (600 ± 21.2 ng As/g), the emunctories (1033 ± 188 ng As/g), and the sciatic nerve (480 ± 50.0 ng As/g) were significantly higher than those derived from other tissues (total 34 tissues, 43.2 ± 2.49 ng As/g) (Fig. 2; Table 1; *p* < 0.0001).

**Table 2:** PAA concentration in cynomolgus monkeys after oral administration of DPAA

	PAA (ng As/g) after administration			
	Days 5 (average ± SEM)	Days 29 (average ± SEM)	Days 170 (average ± SEM)	Days 339 (average ± SEM)
Central nervous system				
Cervical spinal cord	1.92 ± 0.439 (6)	1.14 ± 0.254 (6)	0.196 ± 0.0764 (8)	0.0977 ± 0.0771 (8)
Thoracic spinal cord	1.29 ± 0.442 (6)	0.832 ± 0.269 (6)	0.0668 ± 0.0442 (8)	0.0659 ± 0.0313 (8)
Lumbar spinal cord	2.15 ± 0.496 (6)	0.965 ± 0.261 (6)	0.273 ± 0.0887 (8)	0.0321 ± 0.0208 (8)
Brain stem	2.93 ± 1.47 (3)	1.38 ± 1.05 (3)	0.580 ± 0.214 (4)	0.326 ± 0.00590 (2)
Cerebellum	2.60 ± 1.30 (3)	1.42 ± 0.679 (4)	0.333 ± 0.103 (6)	0 (2)
Frontal lobe	3.13 ± 1.66 (3)	1.61 ± 0.784 (4)	0.358 ± 0.121 (6)	0.302 ± 0.103 (2)
Parietal lobe	3.95 ± 2.16 (3)	1.85 ± 0.796 (4)	0.818 ± 0.239 (6)	0.140 ± 0.0311 (2)
Temporal lobe	2.69 ± 1.71 (3)	1.28 ± 0.794 (4)	0.520 ± 0.188 (6)	0.333 ± 0.332 (2)
Occipital lobe	7.19 ± 5.10 (3)	2.79 ± 0.497 (4)	0.647 ± 0.0913 (6)	0.123 ± 0.122 (2)
Central nervous system (total)	2.77 ± 0.522 (36)	1.40 ± 0.185 (41)	0.391 ± 0.0500 (58)	0.112 ± 0.0288 (36)
Peripheral nervous system				
Sciatic nerve	0.976 ± 0.392 (6)	0.423 ± 0.194 (6)	0 (8)	0 (8)
Emunctories				
Liver	18.0 ± 7.95 (6)	1.84 ± 0.770 (6)	0.0135 ± 0.0126 (8)	0.0157 ± 0.0147 (8)
Gall bladder	20.2 ± 11.7 (4)	1.13 ± 0.653 (4)	0.0200 ± 0.0193 (8)	0 (8)
Kidney	2.69 ± 0.955 (6)	0.482 ± 0.191 (6)	0.0172 ± 0.0163 (8)	0 (8)
Emunctories (total)	12.8 ± 4.36 (16)	1.15 ± 0.351 (16)	0.0170 ± 0.00900 (24)	0.00592 ± 0.00492 (24)
Urogenital system				
Bladder	0.492 ± 0.173 (6)	0.122 ± 0.0570 (6)	0 (8)	0 (8)
Testicle	0.158 ± 0.0908 (4)	0 (5)	0 (4)	0 (4)
Seminal vesicle	0.194 ± 0.193 (4)	0 (5)	0 (4)	0 (4)
Epididymis	0.141 ± 0.140 (4)	0 (5)	0 (4)	0 (4)
Prostate	0.164 ± 0.163 (4)	0 (5)	0 (4)	0 (4)
Ovary	0.235 ± 0.146 (5)	0.100 ± 0.0992 (6)	0.0245 ± 0.0235 (8)	0 (4)
Uterus	0.178 ± 0.119 (5)	0.0253 ± 0.0243 (6)	0 (8)	0 (4)
Vagina	0.165 ± 0.108 (5)	0.0367 ± 0.0357 (6)	0 (8)	0 (4)
Digestive system				
Esophagus	0.355 ± 0.160 (6)	0.0568 ± 0.0559 (6)	0 (8)	0 (8)
Stomach	0.208 ± 0.0954 (6)	0.0311 ± 0.0301 (6)	0 (8)	0 (8)
Duodenum	1.12 ± 0.593 (6)	0.344 ± 0.343 (6)	0 (8)	0 (8)
Small intestine	1.53 ± 1.13 (5)	0.244 ± 0.243 (6)	0 (8)	0 (8)
Colon	0.438 ± 0.214 (6)	0.0424 ± 0.0415 (6)	0 (8)	0 (8)
Rectal	0.144 ± 0.143 (4)	0 (5)	0 (8)	0 (8)
Pancreas	0.384 ± 0.201 (6)	0.0255 ± 0.0246 (6)	0 (8)	0 (8)
Lymph system				
Thymus	0.868 ± 0.398 (6)	0.0752 ± 0.0743 (6)	0 (8)	0 (8)
Spleen	0.655 ± 0.295 (6)	0 (6)	0 (8)	0 (8)
Submandibular node	0.393 ± 0.205 (6)	0 (6)	0 (8)	0.0103 ± 0.00935 (8)
Mesenteric node	0.123 ± 0.122 (4)	0 (6)	0 (8)	0.0153 ± 0.0144(8)
Exocrine system				
Parotid gland	0.327 ± 0.186 (6)	0 (6)	0 (8)	0 (8)
Submandibular gland	0.522 ± 0.297 (6)	0 (6)	0 (8)	0 (8)
Sublingual gland	0.276 ± 0.161 (6)	0 (6)	0 (8)	0 (8)
Lacrimal gland	0.149 ± 0.148 (4)	0 (6)	0 (8)	0 (8)
Endocrine system				
Adrenal glands	0.415 ± 0.188 (6)	0 (6)	0 (8)	0 (8)
Pituitary	0.0469 ± 0.0459 (6)	0 (6)	0 (8)	0 (8)
Thyroid	0.187 ± 0.0658 (6)	0 (6)	0 (8)	0 (8)
Respiratory system				
Trachea	0.141 ± 0.0465 (6)	0 (6)	0 (8)	0 (8)
Lung	1.67 ± 0.552 (6)	0 (6)	0 (8)	0 (8)
Muscular system				
Femoral muscle	0.601 ± 0.193 (6)	0 (6)	0 (8)	0 (8)
Tongue	0.719 ± 0.219 (6)	0 (6)	0 (8)	0.0291 ± 0.0282 (8)
Sense organs				
Eyeball	0.0121 ± 0.0111 (6)	0 (6)	0 (8)	0 (8)
Skin	0.0854 ± 0.0844 (6)	0 (6)	0 (8)	0 (8)
Blood–vascular system				
Heart	0.0330 ± 0.0317 (6)	0 (6)	0 (8)	0.0320 ± 0.0308 (8)
Aorta	0.0483 ± 0.0473 (6)	0 (6)	0 (8)	0 (8)
Other tissues (total)	0.402 ± 0.0537 (186)	0.0339 ± 0.0135 (199)	0.00173 ± 0.000735 (256)	0.00371 ± 0.00147 (244)
Body fluids				
Cerebrospinal fluid	0 (6)	0 (6)	0 (8)	0 (8)
Bile	0 (6)	0 (6)	0 (8)	0 (8)
Hemocyte	0 (6)	0 (6)	0 (8)	0 (4)
Serum	0 (6)	0 (6)	0 (8)	0 (8)

The number between brackets indicates the number of samples examined

**Fig. 3:**
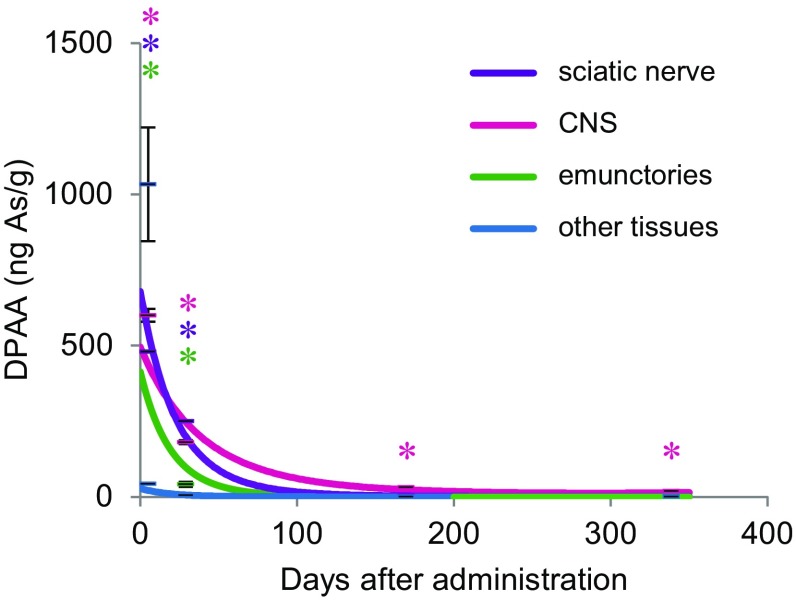
Approximate curves showing the correlation between the days after the last administration of DPAA and the average concentrations of DPAA (ng As/g) in tissue samples from the CNS, the emunctories, the sciatic nerve, and other tissues of cynomolgus monkeys after the daily administration of DPAA (1.0 mg/kg/day) for 28 days. The average values ± SEM for each group are shown using *horizontal lines. Purple*, sciatic nerve; magenta, central nervous system; *green*, emunctories; *blue*, other tissues. *p < 0.0001, in comparison to the other tissues (the *colors* of the *asterisks* correspond to the group)

At 29 days after the last administration, the average concentrations of DPAA in the CNS tissues (181 ± 6.60 ng As/g) and the sciatic nerve (250 ± 23.9 ng As/g) were 30.1 and 52.1% of values at the 5-day time point, respectively (Fig. 3; Table 1). On the other hand, the average concentrations of DPAA in the emunctories (41.6 ± 8.32 ng As/g) and other tissues (4.76 ± 0.311 ng As/g) were drastically lower in comparison to those of the group that was euthanized at 5 days after the last administration (Fig. 3; Table 1) (4.03 and 11.0% of values at the 5-day time point, respectively). We also examined the half-life of DPAA in each tissue (Table 1). The average half-lives of DPAA in the CNS tissues and the sciatic nerve (14.4 and 25.7 days, respectively) were significantly longer in comparison to DPAA in other tissues (*p* < 0.0001).

In the group that was euthanized at 339 days after the last administration, the concentrations of DPAA in all of the tissues were more than 96% lower than those in the group euthanized at 5 days after the last administration (Table 1). Notably, the average concentration of DPAA in the CNS tissues (18.8 ± 1.48 ng As/g) was significantly higher than that in all of the other tissues, even at 339 days after the last administration (Fig. 3; Table 1; *p* < 0.0001).

### Quantitative analysis of DPAA in the body fluids of cynomolgus monkeys

**Fig. 4:**
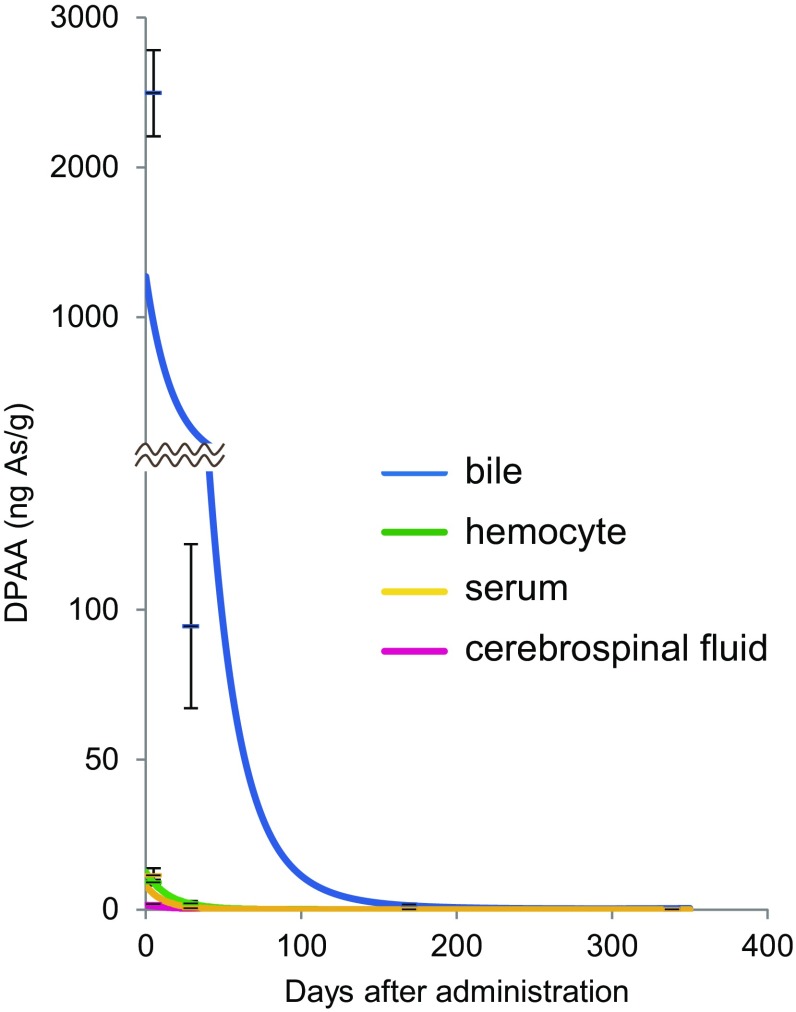
Approximate curves showing the correlation between the days after the last administration of DPAA and the average concentrations of DPAA (ng As/g) in the body fluids (cerebrospinal fluid, bile, hemocyte and serum) of cynomolgus monkeys after the daily administration of DPAA (1.0 mg/kg/day) for 28 days. *Blue*, bile; *green*, hemocyte; *yellow*, serum; magenta, cerebrospinal fluid

Next, we measured the concentration of DPAA in the body fluids of each group using an LC–MS analysis. A small amount of DPAA was detected in the serum and the hemocytes at 5 and 29 days after the last administration (Fig. 4; Table 1). It was not detected after these time points. Similarly, DPAA was detected at a concentration of 0.233 ± 0.0268 ng As/g in the CSF at 29 days, and was not detected later (Fig. 4; Table 1). On the contrary, a large amount of DPAA was detected in the bile at 5 days after the last administration (2496 ± 287 ng As/g) (Table 1); the concentration at 29 days after the last administration was 3.79% of the value at the 5-day time point (94.5 ± 27.4 ng As/g). The average half-life of DPAA in the bile was 4.88 days.

### Quantitative analysis of PAA and PMAA in cynomolgus monkeys

PAA and PMAA are known to be the major metabolites of DPAA. We measured the concentrations of PAA and PMAA in the same tissues of each group described above using an LC–MS analysis. We detected very small amounts of PAA and PMAA in these tissues at 5–339 days after the last administration (Tables 2, 3). The concentrations of PAA and PMAA in the CNS tissues (2.77 ± 0.522 and 0.973 ± 0.184 ng As/g, respectively) and emunctories (12.8 ± 4.36 and 1.66 ± 0.658 ng As/g, respectively) were a little higher than those in other tissues at 5 days after the last administration (Tables 2, 3).

**Table 3:** PMAA concentration in cynomolgus monkeys after oral administration of DPAA

	PMAA (ng As/g) after administration			
	Days 5 (average ± SEM)	Days 29 (average ± SEM)	Days 170 (average ± SEM)	Days 339 (average ± SEM)
Central nervous system				
Cervical spinal cord	0.592 ± 0.204 (6)	0.380 ± 0.177 (6)	0.229 ± 0.113 (8)	0.0304 ± 0.0294 (8)
Thoracic spinal cord	0.511 ± 0.254 (6)	0.416 ± 0.183 (6)	0.226 ± 0.0981 (8)	0.0272 ± 0.0262 (8)
Lumbar spinal cord	0.599 ± 0.284 (6)	0.387 ± 0.156 (6)	0.196 ± 0.100 (8)	0.0465 ± 0.0455 (8)
Brain stem	1.86 ± 0.460 (2)	1.61 ± 0.370 (3)	0.146 ± 0.145 (4)	0 (8)
Cerebellum	2.16 ± 1.29 (2)	0.741 ± 0.377 (4)	0.199 ± 0.0755 (6)	0.166 ± 0.165 (2)
Frontal lobe	1.54 ± 0.896 (2)	0.523 ± 0.312 (4)	0.0524 ± 0.0514 (6)	0 (8)
Parietal lobe	2.62 ± 1.20 (2)	0.515 ± 0.312 (4)	0.0690 ± 0.0689 (6)	0 (8)
Temporal lobe	0.497 ± 0.496 (2)	0.143 ± 0.142 (4)	0 (8)	0 (8)
Occipital lobe	0.813 ± 0.812 (2)	0.454 ± 0.264 (4)	0.0601 ± 0.0591 (6)	0 (8)
Central nervous system (total)	0.973 ± 0.184 (30)	0.523 ± 0.0899 (41)	0.139 ± 0.0299 (58)	0.0326 ± 0.0156 (36)
Peripheral nervous system				
Sciatic nerve	0.0391 ± 0.0381 (6)	0.124 ± 0.123 (4)	0 (8)	0 (8)
Emunctories				
Liver	3.77 ± 1.55 (6)	1.60 ± 0.430 (6)	0.0666 ± 0.0372 (8)	0.0762 ± 0.0437 (8)
Gall bladder	0.543 ± 0.329 (5)	0.112 ± 0.0639 (4)	0.0280 ± 0.0270 (8)	0.0225 ± 0.0215 (8)
Kidney	0.476 ± 0.244 (6)	0.285 ± 0.106 (6)	0.0608 ± 0.0598 (8)	0.0124 ± 0.0114 (8)
Emunctories (total)	1.66 ± 0.658 (17)	0.736 ± 0.235 (16)	0.0518 ± 0.0243 (24)	0.0371 ± 0.0170 (24)
Urogenital system				
Bladder	0.181 ± 0.0911 (6)	0.0820 ± 0.0810 (4)	0 (8)	0 (8)
Testicle	0.228 ± 0.160 (5)	0 (8)	0 (8)	0 (8)
Seminal vesicle	0.145 ± 0.0953 (5)	0 (8)	0 (8)	0 (8)
Epididymis	0.0997 ± 0.0634 (5)	0 (8)	0 (8)	0 (8)
Prostate	0.180 ± 0.127 (5)	0 (8)	0 (8)	0 (8)
Ovary	0.0753 ± 0.0743 (4)	0.0683 ± 0.0673 (4)	0 (8)	0 (8)
Uterus	0.0722 ± 0.0712 (4)	0.0743 ± 0.0733 (4)	0 (8)	0 (8)
Vagina	0.0264 ± 0.0254 (4)	0.0912 ± 0.0902 (4)	0 (8)	0 (8)
Digestive system				
Esophagus	0.178 ± 0.0935 (6)	0.0799 ± 0.0789 (4)	0 (8)	0 (8)
Stomach	0.168 ± 0.0837 (6)	0.0536 ± 0.0526 (4)	0 (8)	0 (8)
Duodenum	0.218 ± 0.107 (6)	0.152 ± 0.0683 (6)	0 (8)	0 (8)
Small intestine	0.211 ± 0.110 (6)	0.102 ± 0.0544 (6)	0 (8)	0 (8)
Colon	0.130 ± 0.0625 (6)	0.801 ± 0.711 (6)	0 (8)	0 (8)
Rectal	0.0699 ± 0.0689 (5)	0 (8)	0 (8)	0 (8)
Pancreas	0.278 ± 0.175 (6)	0.149 ± 0.107 (4)	0 (8)	0 (8)
Lymph system				
Thymus	0.211 ± 0.140 (6)	0.0821 ± 0.0656 (4)	0 (8)	0 (8)
Spleen	0.267 ± 0.170 (6)	0.168 ± 0.106 (4)	0 (8)	0 (8)
Submandibular node	0.141 ± 0.0895 (6)	0.528 ± 0.403 (5)	0 (8)	0 (8)
Mesenteric node	0.0924 ± 0.0914 (5)	1.41 ± 1.12 (4)	0 (8)	0 (8)
Exocrine system				
Parotid gland	0.178 ± 0.113 (6)	0.134 ± 0.0917 (4)	0 (8)	0 (8)
Submandibular gland	0.305 ± 0.207 (6)	0.168 ± 0.120 (4)	0 (8)	0 (8)
Sublingual gland	0.169 ± 0.108 (6)	0.105 ± 0.0703 (4)	0 (8)	0 (8)
Lacrimal gland	0.100 ± 0.0990 (5)	0 (8)	0 (8)	0 (8)
Endocrine system				
Adrenal glands	0.434 ± 0.276 (6)	0.487 ± 0.415 (5)	0 (8)	0 (8)
Pituitary	0.146 ± 0.101 (6)	0.125 ± 0.0863 (4)	0 (8)	0 (8)
Thyroid	0.582 ± 0.406 (6)	0.858 ± 0.298 (6)	0 (8)	0 (8)
Respiratory system				
Trachea	0.0563 ± 0.0355 (6)	0.0662 ± 0.0652 (4)	0 (8)	0 (8)
Lung	0.345 ± 0.157 (6)	0.177 ± 0.0610 (6)	0.0267 ± 0.0257 (8)	0 (8)
Muscular system				
Femoral muscle	0.305 ± 0.133 (6)	0.101 ± 0.0802 (4)	0.0276 ± 0.0266 (8)	0 (8)
Tongue	0.197 ± 0.0872 (5)	0.143 ± 0.112 (4)	0.0716 ± 0.0574 (8)	0 (8)
Sense organs				
Eyeball	0 (8)	0 (8)	0 (8)	0 (8)
Skin	0.431 ± 0.392 (6)	0 (8)	0 (8)	0 (8)
Blood–vascular system				
Heart	0.217 ± 0.114 (6)	0 (8)	0 (8)	0 (8)
Aorta	0 (8)	0 (8)	0 (8)	0 (8)
Other tissues (total)	0.199 ± 0.0268 (186)	0.207 ± 0.0514 (145)	0.00462 ± 0.00205 (272)	0 (8)
Body fluids				
Cerebrospinal fluid	0 (8)	0 (8)	0 (8)	0 (8)
Bile	0 (8)	0 (8)	0 (8)	0 (8)
Hemocyte	0 (8)	0.0161 ± 0.0151 (6)	0 (8)	0 (8)
Serum	0 (8)	0.00523 ± 0.00423 (6)	0 (8)	0 (8)

The number between brackets indicates the number of samples examined

## Discussion

In the present study, we examined the clearance of orally administrated DPAA in cynomolgus monkeys. Our results clearly demonstrated that the half-life of DPAA in the brain was twice as long as that in other tissues except the emunctories, suggesting that DPAA easily accumulates in the brains of cynomolgus monkeys. Together with our preliminary observation that the half-life of DPAA in the rat brain after the administration of DPAA (at the same dose as this study) was shorter than that of cynomolgus monkeys (<8.6 days; Masuda and Ishii, unpublished observation), the findings suggest that DPAA may accumulate in the brains of primates more easily than in the rodent brain. Previous experiments using rodents have shown that inorganic arsenic (iAs) can cross the blood–brain barrier (BBB; Rodríguez et al. 2005; Juárez-Reyes et al. 2009). Similarly to organic arsenic, DPAA was also detected in the CSF obtained from the residents of Kamisu who were exposed to DPAA (Ishii et al. 2014). Their CSF/serum ratios of DPAA were 3.7 and 3.0% at the first and 14th days after their last exposure, respectively (Ishii et al. 2014). Consistent with these previous results, DPAA was detected in the CSF of cynomolgus monkeys in this study. The average CSF/serum ratio of DPAA in this study was 15.7% (5 days after the last administration, *n* = 6). Thus, these results strongly suggest that DPAA may pass through the BBB of primates. This may be due to the hydrophobicity of DPAA (Weksler et al. 2005; Naranmandura et al. 2009). Also, the reduction in blood flow and the cerebral metabolism was observed in the brains of the Kamisu residents who were exposed to DPAA (unpublished data). This evidence strongly suggests that the long presence of DPAA in the human brain may affect the brain function.

Several previous studies have shown that small amounts of iAs, monomethylarsenous acid (MMA) and dimethylarsinic acid (DMA) accumulated in the rat brain after the administration of iAs (Rodríguez et al. 2005, 2010; Juárez-Reyes et al. 2009). In humans, keratinization and pigmentation of the skin, peripheral neuropathy, skin cancer, and peripheral circulatory failure have all been reported as symptoms of chronic poisoning from exposure to inorganic arsenic compounds; however, there have been few reports on CNS symptoms (World Health Organization 2001). Previous rodent studies have revealed that DPAA, an organic arsenic compound, accumulated in the rodent brain more easily than inorganic arsenic compounds (Naranmandura et al. 2009; Ozone et al. 2010). Our study also demonstrated that DPAA accumulated in the CNS tissues of primates for a long period after its oral administration. These facts are consistent with our previous observations that the effects of DPAA on humans were substantially restricted to regions of the CNS such as the cerebellum and brain stem (Ishii et al. 2004). Furthermore, the cerebellar dysfunction found in the recent animal behavior experiments was quite similar to that observed in the Kamisu residents who were exposed to DPAA (Ozone et al. 2010; Negishi et al. 2013). Thus, it is highly likely that the effects of DPAA exposure on the CNS of Kamisu residents were caused by its long-term accumulation in the CNS.

It is known that when DPAA is administered to rats, it is excreted in the urine, feces, and hair (Naranmandura et al. 2009; Kobayashi and Hirano 2013). Although a high level of DPAA accumulation was seen in the liver, gallbladder, and bile of cynomolgus monkeys at 5 days after the last DPAA administration, the half-lives of the DPAA in these tissues were as short as those in other tissues. Thus, as is the case with rats, DPAA is likely to be excreted in the liver, gallbladder, and bile of cynomolgus monkeys. While the concentration of DPAA in rat hair at 14 days after the last DPAA administration (at the same dose as this study) was high (Masuda and Ishii, unpublished observation), moderate levels of DPAA and its metabolites were also detected in the skin with hair of cynomolgus monkeys at 5 days after the last DPAA administration in the present study. Thus, like the rat, it is highly likely that DPAA and its metabolites may be excreted in skin with hair in cynomolgus monkeys.

In summary, we showed, for the first time, that orally administered DPAA accumulated in the CNS tissues of primates. This finding could contribute to future studies to investigate the dynamics and metabolism of DPAA in humans.
